# Relationship between Cardiorespiratory Fitness and Executive Function in Young Adults: Mediating Effects of Gray Matter Volume

**DOI:** 10.3390/brainsci12111441

**Published:** 2022-10-26

**Authors:** Yuexin Liu, Lina Zhu, Kelong Cai, Xiaoxiao Dong, Xuan Xiong, Zhimei Liu, Aiguo Chen

**Affiliations:** 1 College of Physical Education, Yangzhou University, Yangzhou 225127, China; 2 Institute of Sports, Exercise and Brain, Yangzhou University, Yangzhou 225127, China

**Keywords:** cardiorespiratory fitness, executive function, gray matter volume, young adults, mediation effect

## Abstract

We evaluated the association between cardiorespiratory fitness (CRF) and executive function (EF) in young adults and the mediating effects of GMV on this relationship. This study involved 217 college students. An incremental load exercise program was used to evaluate VO_2_max. EF was estimated by the Flanker task, the 2-back task, and the more-odd shifting task, while structural magnetic resonance and region-based morphometry (RBM) were used to analyze GMV. The high CRF group had a shorter updating reaction time (RT) (*p* ≤ 0.05). CRF was positively correlated with the right orbital part of the middle frontal gyrus (ORBmid.R) GMV (*p* ≤ 0.05). ORBmid.R GMV was negatively correlated with updating RT (*p* ≤ 0.05). Model 4 in SPSS was used to assess the mediating effects of ORBmid.R GMV between CRF and updating RT. ORBmid.R GMV was established to have a partially mediating role between CRF and updating RT, which accounted for 19.6% of the total effect value. These findings indicate that the negative correlation between CRF and EF was significant, and ORBmid.R GMV played a mediating role in the relationship between CRF and EF, providing new evidence toward comprehensively revealing that CRF promotes EF performance.

## 1. Introduction

Throughout life, brain and cognition health among adolescents and young adults can influence academic achievement and overall health [1,2], which necessitates the identification of the predictors and modifiers of brain health at a young age [3]. Due to advances in the social economy, there has been a growing interest in the influence of lifestyle factors, such as regular physical exercise, in the promotion of a relatively high level of cardiorespiratory fitness (CRF) and health (e.g., brain health) [2,4,5,6]. CRF is operationalized by maximal oxygen consumption (VO_2_max) and is correlated with the functions of different physiological systems [7,8,9]. It affects the development of executive function (EF). Increasing physical activity levels and improving CRF are beneficial for the healthy development of EF [10,11]. Low levels of CRF in early adulthood are associated with higher risks of cardiovascular disease in late life [12,13,14] and negatively affect psychological functions (resulting in depression and anxiety) and EF, becoming evident in accelerated cognitive decline and brain atrophy in later years [15,16,17,18,19].

Physiologically, EF plays a pivotal role in cognitive functions [20], including: (i) inhibition (i.e., resisting habits, temptations, or distractions); (ii) updating (i.e., retaining and using information); and (iii) cognitive flexibility [21,22]. Various neuropsychological paradigms, such as the Flanker task, the 2-back task, and the more-odd shifting task, can be used to measure the performance of EF. Improvements in CRF have been shown to effectively enhance children’s working memory, further modifying their cognitive flexibility [23,24,25] and optimizing inhibition control among the elderly to slow down the degradation of visuospatial memory functions due to aging [26,27,28]. High CRF and physical activity (PA) levels have been consistently associated with the maintenance of cognitive functions in life, including a reduced risk of developing Alzheimer’s disease and a slow progression of cognitive problems in cognitively impaired patients [29,30,31].

However, the relationship between CRF and EF among adults has not been conclusively established [32]. Findings from previous studies [33,34,35] are inconsistent, which may be due to the big age gap among study participants. Therefore, it is necessary to comprehensively assess the relationship between CRF and the three sub-functions of young adults’ EF.

Advances in brain imaging technologies have facilitated the evaluation of underlying physiological mechanisms involved in the relationship between CRF and EF. Structural plasticity features of the brain mediate this relationship. At any age, CRF has important effects on human brain health [36] and acts as a protective factor against gray matter atrophy among the elderly [32,37,38,39,40,41]. There is a positive association between CRF and gray matter volume (GMV), especially among the elderly [42,43,44,45,46,47]. Elevated levels of CRF are associated with increased hippocampal and prefrontal cortex volume as well as better cognitive performance among the elderly [48]. CRF and cortical volume (i.e., frontal cortex), as well as EF (i.e., updating), are positively correlated among children [47,49,50].

College students are in the early adulthood phase in which the GMV in each brain area gradually increases and can effectively predict the development of its EF. The larger the GMV in the frontal lobe region, the better the EF [51]. Most studies have focused on children and older adults, while younger adults have not been assessed [52]. The relationships between CRF, brain structure, and EF, which have not been fully described in younger adults, are necessary for the assessment of exercise–cognition interactions [53]. We hypothesized that GMV plays an intermediary role in the influence of CRF on EF.

Based on the available scientific evidence, our hypotheses are: GMVs are used as intermediary variables for the effect of CRF on EF to construct an intermediary mode. We reveal that GMV is a potential neural pathway through which CRF affects an individual’s EF and provides a new perspective for the comprehensive understanding of the relationship between CRF and EF.

## 2. Materials and Methods

### 2.1. Participants

This study involved 221 freshmen aged between 18–20 years from Yangzhou University. The participants came from comparable sociocultural environments and followed a commonly prescribed syllabus as well as examination evaluation patterns. Before testing, participants were only asked to indicate whether they had sports habits rather than specific sports events and durations in their lives. The inclusion criteria were: (i) no history of mental or genetic disorders; (ii) test visual acuity or corrected visual acuity > 0.8, no color blindness or color deficiency; (iii) no serious physical illness, no history of brain trauma or nervous system disease, and no history of drug and alcohol dependence or other diseases that may affect the structure and function of the brain; (iv) right-handed; (v) college students with abnormal intelligence, as revealed by Raven’s Standard Progressive Matrices (SPM) test, were excluded; (vi) participants who met the conditions for magnetic resonance scanning, such as the absence of implanted metals (including metal dentures, etc.) and electronic, magnetic, or mechanical equipment (such as pacemakers) in the body. According to the above criteria, 4 participants were knocked out (3 males with MRI data missing and 1 female who exited). Our pooled dataset eventually included 217 participants (including 98 males and 119 females). The experiment was conducted in Yangzhou, China, with approval from the Ethical and Human Protection Committee of the Affiliated Hospital of Yangzhou University (2017-YKL045-01). Participants signed an informed consent form. All study procedures were in accordance with the latest version of the Declaration of Helsinki.

### 2.2. Cardiorespiratory Fitness Testing

VO_2_max was tested using an increasing load exercise protocol [54], i.e., CRF. Elmed EGT 1000 was used to increase the load, while VO_2_max was measured using the Cortex Metalyze R-II benchtop gas metabolism analyzer (Germany). The increasing load exercise scheme was as follows: starting load was 50 w, treadmill rhythm was 55~60 r/min, and increment was 50 w every 3 min until exhaustion.

Before the test, all participants completed a physical activity preparation questionnaire to ensure that they had not performed high-intensity exercises the day before nor taken drugs or drinks that were stimulating or inhibiting the nerves. They were prepared for 3 to 5 min before the formal test to prevent sports-associated injuries. The basal heart rate was measured, after which exercises were started when the heart rate returned to a quiet state. A polar heart rate band was used to monitor changes in heart rate during the exercise. All participants who met any of the following four indicators achieved VO_2_max: (i) with increasing load, oxygen uptake remained unchanged or slightly decreased (1500 mL/min); (ii) respiratory quotient >1.1; (iii) HR > 180 b/min; (iv) despite repeated encouragement, participants could not maintain a cycling rate of 55 to 60 r/min. After the completion of the test, VO_2_max was recorded, followed by resting for about 5 min. In the case that the participants’ bodies had no abnormal reactions, they were left alone. CRF was obtained by multiplying the VO_2_max value (L/kg/min) by 1000 and dividing it by body weight (kg).

As previously reported [55], participants with CRF < 30% were assigned to the low CRF group (65 people; 29 males and 36 females), while those with CRF > 70% were assigned to the high CRF group (65 people; 39 males and 26 females).

### 2.3. Executive Function Assessment

In this study, the Flanker task, the 2-back task, and the more-odd shifting task were used to evaluate updating, inhibition, and cognitive flexibility, respectively. The test tool has high reliability and validity and is unanimously recognized by peer experts [25]. Test indices were reaction time/ms and accuracy rate/%. The shorter the reaction time, the higher the operation efficiency of the function. The higher the accuracy rate, the better the function performance.

#### 2.3.1. Flanker Task

The Flanker task was assessed by modified Eriksen [25]. In brief, a range of English letters appeared on the screen under congruent or incongruent conditions. Participants needed to discriminate the letter in the middle of the screen as soon as they could by pressing the “F” or “L” keys. The two conditions were equally represented and randomly presented. The test was made of two parts, and each part contained 48 trials, in which the duration of letter presentation was 1000 ms, the stimulation interval was 2000 ms, and the maximal reaction time was 2000 ms. If participants did not finish within 2000 ms, the trial RT was still recorded as 2000 ms. 

#### 2.3.2. 2-Back Task

The 2-back task [56,57,58] was designed to assess updating. Briefly, a series of numbers would appear on the screen (i.e., 1, 2, 3, and 4). Each 2-back test was composed of 13 figures in a random sequential lineup. The participants were asked to remember the second and third numerals in the sequence of appearance. When the fourth stimulus appeared, they needed to judge whether it was the same as the second by pressing the “A” key for yes or the “B” key for no with both hands on the keyboard. The stimulation interval was 2000 ms and the maximal reaction time was 2000 ms. If participants did not finish within 2000 ms, the trial RT was still recorded as 2000 ms.

#### 2.3.3. More-Odd Shifting Task

The more-odd shifting task was assessed by modified Hambrick [59]. In a nutshell, a series of numbers from either 1 to 4 or 6 to 9 would appear on the screen. Each more-odd shifting task consisted of 3 parts. The A part involved 16 homogeneous trials in which the numbers were printed on the screen. Participants used their left or right finger to indicate whether the presented number was greater than or less than 5 by pressing the “F” or “L” keys, respectively. The B part also involved 16 homogeneous trials in which green numbers were presented on the screen. Participants used their left or right finger to indicate whether the presented number was odd or even by pressing the “F” or “L” keys, respectively. The C part involved 32 homogeneous trials in which the numbers printed on the screen were from both the A and B trials. Participants needed to identify if the presented number was greater or less than 5 in black and if the presented number was odd or even in green. Pressing the wrong button and failing to respond within 150–1000 ms for homogeneous trials or within 300–1500 ms for heterogeneous trials were considered incorrect responses.

The stimulation interval was 2000 ms, and the stimulus-onset asynchrony was 2000 ms. The shifting index used in this study was the global switch cost, which was calculated as differences in response time between heterogeneous (i.e., the average of the C parts) and homogeneous (i.e., the average of the A and B parts) blocks.

### 2.4. MRI Data Acquisition

#### 2.4.1. T1-Weighted Image Data Acquisition

GE Discovery MR750W 3.0 T magnetic resonance imaging was used for image acquisition. The T1-MPRAGE sequence structure image scan parameters were: TR/TE = 7.20/3.06 ms, TI = 450 ms, slice thickness = 1.00 mm, flip angle = 12°, acquisition matrix = 256 × 256, and field of view = 256 × 256 mm.

#### 2.4.2. GMV Data Pre-Processing

The MRI data were processed using SPM12 implemented in MATLAB. SPM12 was originally used to analyze the MRIs obtained from all participants and gross anatomical abnormalities and to exclude artifacts. The MRIs were artificially adjusted to the anterior commissure to raise registration. Then, every MRI was divided into 3 parts (i.e., gray matter, white matter, and cerebrospinal fluid) by employing the toolbox function [56]. Finally, an 8 mm full-width-at-half-maximum Gaussian kernel was used to smooth the regulated images and to enhance the quality of the signal-to-noise ratio. The rex plug-in was used to extract the GMV of significantly changed areas. The threshold was set at *p* < 0.05, the voxel threshold at *p* < 0.01, and the cluster size at >50 voxels with FDR correction.

### 2.5. Statistical Analysis

The Statistical Package for Social Sciences (SPSS; SPSS Inc., Chicago, IL, USA) version 26.0 for Windows was used for the analyses. The chi-square test and the independent sample t-test were used to compare differences in the demographic variables and the CRF and EF of the high and low CRF groups, while a multivariate analysis of variance (MANOVA) was used to investigate differences between GMVs of the two groups; on this basis, brain regions with significant differences in GMV were further assessed. Using partial correlation analysis to control gender, age, and BMI, correlations between CRF, GMV (brain area with significant differences), and EF were used in process plug-in Model 4 to test the mediating effects of GMV on the relationship between CRF and EF. The deviation-corrected percentile bootstrap method was used to estimate 95% confidence intervals of the mediating effects by sampling 5000 bootstrap samples. *p* ≤ 0.05 was the threshold for significance.

## 3. Results

### 3.1. Demographics, CRF, and EF of the Two Groups

The gender, age, and BMI of college students affect their EF [57,58]. Therefore, we controlled for the above variables and the gender of the two groups (χ^2^ = 0.050, *p* > 0.05). The independent sample *t*-test was used to analyze differences in age, BMI, VO_2_max, relative VO_2_max, and EF subdomain. The obtained results indicated that there were significant differences in BMI (t(128) = 3.443, *p* ≤ 0.001), VO_2_max (t(128) = −19.587, *p* ≤ 0.001), relative VO_2_max (t(128) = −34.014, *p* ≤ 0.001), inhibition RT (t(128) = 2.356, *p* ≤ 0.05), updating RT (t(128) = 2.473, *p* ≤ 0.05), cognitive flexibility RT (t(128) = 6.514, *p* ≤ 0.001), and updating accuracy rate (t(128) = −2.344, *p* ≤ 0.05) between the two groups. However, there were insignificant differences in the inhibition accuracy rate (t(128) = −0.798, *p* > 0.05) and cognitive flexibility accuracy rate (t(128) = −0.693, *p* > 0.05), indicating that the high CRF was only better in updating (Table 1). The Kolmogorov–Smirnov test was used to assess normal distribution in sub-EF RT (*p* > 0.05). The numbers over third standard deviations were regarded as outliers [59]. The split-half reliability test also showed the significant trustworthiness of the Flanker task (r = 0.964) and the more-odd shifting task (r = 0.666).

### 3.2. Effects of CRF on the GMV of the Two Groups

**Figure 1:**
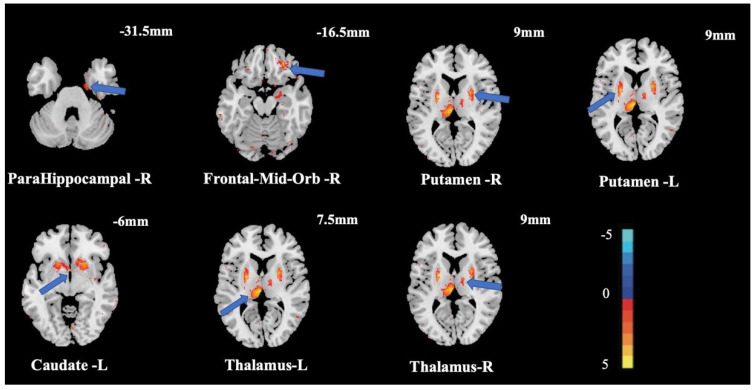
Changes in GMV between groups. Note: Numbers in the figure represent coordinates of this section in the vertical axis (Z-axis); The light band and numbers in the lower right corner show correspondence between light and dark areas in the brain.

The stochastic effect model of SPM12 was used to analyze the GMV of the two groups and to explore the relationship between CRF and GMV. The obtained results indicated that there were significant differences in the right frontal-mid-orb (ORBmid.R), right parahippocampal (PHG.R), left caudate (CAU.L), left putamen (PUT.L), right putamen (PUT.R), left thalamus (THA.L), and right thalamus (THA.R) (Table 2, Figure 1). All results were corrected by FDR (*p* = 0.05).

BMI is a covariate that should be included in the assessment of the impact of CRF on GMV in ROI. A multivariate analysis of variance analysis showed that there were significant differences (Wilks’ lambda = 0.834, F(8,121) = 3.018, *p* ≤ 0.05) between the groups. Between-group analyses revealed significant differences in ORBmid.R GMV (Wilks’ lambda = 0.881, F(2,127) = 8.596, *p* ≤ 0.05). For multiple corrections, Bonferroni corrections were applied (α = 0.014). Based on this analysis, we found that a high CRF increases ORBmid.R GMV (Table 3).

### 3.3. Analysis of CRF, GMV, and EF

**Figure 2:**
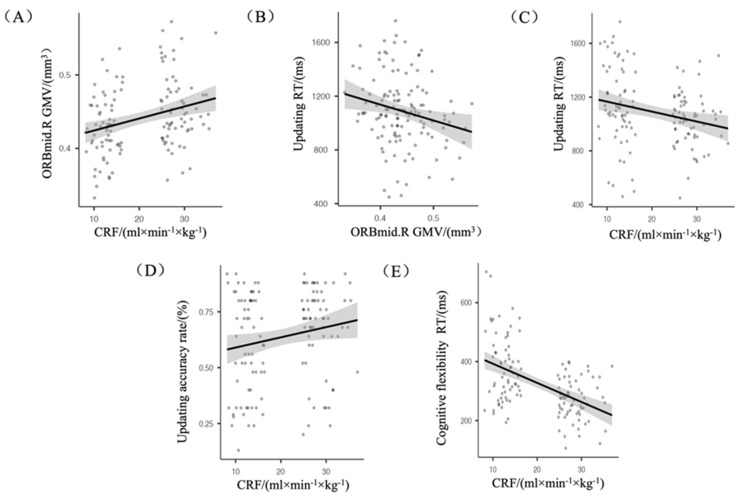
(**A**) CRF was positively correlated with ORBmid.R GMV; (**B**) ORBmid.R GMV was negatively correlated with updating RT; (**C**) CRF was negatively correlated with updating RT; (**D**) CRF was positively correlated with the updating accuracy rate; (**E**) CRF was negatively correlated with cognitive flexibility RT.

Age, sex, and BMI were used as controls for partial correlation analysis of correlations between CRF, GMV, and each sub-function of ACC and RT. It was found that: (i) CRF was positively correlated with ORBmid.R GMV (r = 0.230, *p* ≤ 0.05) (Figure 2A); (ii) ORBmid.R GMV was negatively correlated with updating RT (r = −0.186, *p* ≤ 0.05) (Figure 2B); and (iii) CRF was negatively correlated with updating RT (r = −0.184, *p* ≤ 0.05) (Figure 2C), positively correlated with updating accuracy rate (r = 0.202, *p* ≤ 0.05) (Figure 2D), and positively correlated with cognitive flexibility RT (r = −0.490, *p* ≤ 0.001) (Figure 2E).

### 3.4. Mediating Role of GMV between CRF and Updating RT

**Figure 3:**
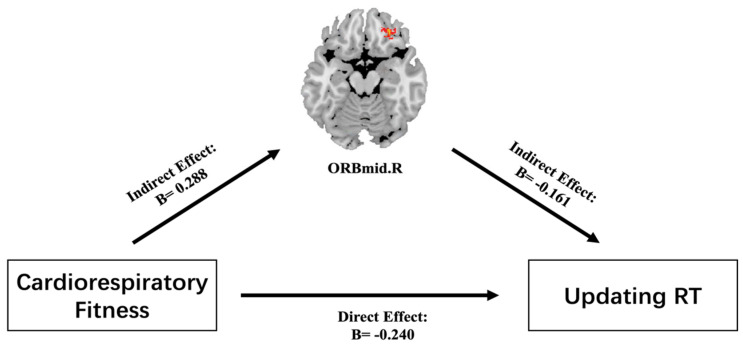
Mediating effect of ORBmid.R GMV on the relationship between CRF and updating RT. Note: The red area is ORBmid.R GMV, while the values are standardized β coefficients for the mediating test.

There were significant correlations between CRF, ORBmid.R GMV, and updating RT. Model 4 (simple mediating model) in the Hayes SPSS macro was used to control for sex, age, and BMI, after which the mediating effects of CRF, ORBmid.R GMV, and updating RT were assessed. It was found that CRF had significant predictive effects on updating RT (B = −0.240, t = −2.793, *p* ≤ 0.05). After the insertion of the mediating variable, the direct predictive effect of CRF on updating RT was still significant (B = −0.193, t = −2.177, *p* ≤ 0.05). The positive predictive effects of CRF and ORBmid.R GMV were significant (B = 0.288, t = 3.400, *p* ≤ 0.05), and the negative predictive effect of ORBmid.R GMV on updating RT was also significant (B = −0.161, t = −1.814, *p* ≤ 0.05). The upper and lower limits of the bootstrap 95% confidence interval for the effects of CRF and updating RT and the mediating effect of ORBmid.R GMV did not contain 0. This shows that CRF can directly predict updating RT and can also predict updating RT through the mediating effects of ORBmid.R GMV. These direct (−0.193) and mediating (−0.047) effects respectively accounted for 80.4% and 19.6% of the total effect (−0.240) (Table 4 and Table 5, Figure 3).

## 4. Discussion

ORBmid.R GMVs are significant mediators in the link between CRF and updating RT, which validates our hypothesis. The higher the CRF, the shorter the updating RT, indicating that CRF has a significant negative predictive effect on updating RT among college students. The direct effect size was 80.4%. Previous studies on the relationship between CRF and EF showed that higher CRF tended to higher efficiency in the frontal parietal network. Meanwhile, higher efficiency in the frontal parietal network was associated with superior EF. Therefore, the relationship between CRF and EF can be adjusted by having better efficiencies in the frontal parietal network of people.

This study further used PROCESS to establish the mediating effect based on correlations among CRF, ORBmid.R GMV, and updating. It was established that the ORBmid.R GMV of college students played a partial mediating role between CRF and updating. First, higher CRF resulted in bigger ORBmid.R GMV, thereby affecting neural connections in areas such as the frontal lobe and improving the activities of underlying neural networks, which might lift the performance of EF. Second, a bigger ORBmid.R GMV exerts direct effects on their receptors within the prefrontal cortex and hippocampus and improves the performance of EF, at least in part via those mechanisms. In this study, ORBmid.R GMV was responsible for 19.6% of the CRF-associated effects on updating. This indicates that CRF affects updating through ORBmid.R GMV. Therefore, CRF-related ORBmid.R GMV plays a role in CRF-related updating.

Differently, CRF was positively correlated with motor area GMV (i.e., putamen, caudate, thalamus, and parahippocampal regions). However, it was not found that the above four regions were related to the three sub-functions of EF. The putamen, caudate, and thalamus are the most concentrated parts of motor conduction and integration coordination, and they play an important role in CRF but are less related to EF [60]. The parahippocampal region may be the only one of four regions associated with EF. Previous research has reported that compared with the elderly, parahippocampal GMV is positively correlated with EF. It is stronger in the elderly than in young adults [61,62,63]. We have not come to the same conclusion in this study, which may be related to lower direct correspondence between the parahippocampal GMV and the EF of college students in early adulthood.

The mediation model was used in our study for the first time to investigate the mediating roles of ORBmid.R GMV in the relationship between CRF and EF, and the potential neural pathways of this relationship were found. Nevertheless, this study is limited by several factors. First, despite the large sample size, this study only covers the effects of CRF on GMV and EF in young and fit healthy adults. Second, the study only collects BMI data; it is important for us to control for obesity using other indexes such as waist-to-hip ratios in later life. Finally, the study mainly understands the relationship between CRF and EF from cerebral cortex development but not by analyzing serum concentration. We look forward to uncovering the association between CRF and EF from the molecular perspective in the future. Our findings are consistent with the theory that CRF is important in EF. Several studies have revealed associations between CRF and EF, implying that enhanced EF promotes decision-making with regard to exercise, thereby contributing to healthy outcomes.

## 5. Conclusions

Using a sample drawn from a study of college students, we found that higher CRF was related to increased ORBmid.R GMV, which was itself related to shorter updating RT. We examined this potential role and found that ORBmid.R GMV mediated the association between CRF and EF. CRF-related ORBmid.R GMV may be a new pathway underpinning the link between CRF and EF. These findings suggest that even in younger adults, CRF can predict neurological differences leading to structural brain changes, such as greater volume in the ORBmid.R, which may, in turn, forecast a more active and efficient use of EF. Therefore, a future study in this area will be essential in developing more effective exercise programs for the wider population and exploring the causal relationship between CRF and EF on different levels (e.g., brain, behavior). This may help establish a scientific and effective theoretical basis and core technology for the formulation of exercise intervention programs for the coordinated development of “body, mind, and brain”.

## Figures and Tables

**Table 1:** Participants’ demographics, cardiorespiratory fitness, and executive function (M ± SD).

Variable	Low CRF Group	High CRF Group
Number	65 (Female 36)	65 (Female 26)
Age	19.307 ± 0.634	19.12 ± 0.516
BMI/(kg × m^−2^)	22.834 ± 3.869 ***	20.85 ± 2.552 ***
VO_2_max/(L × kg^−1^ × min^−1^)	0.791 ± 0.201 ***	1.75 ± 0.345 ***
Relative VO_2_max/(mL × min^−1^ × kg^−1^)	12.598 ± 2.166 ***	28.43 ± 3.064 ***
Inhibition		
RT/ms	19.393 ± 19.531 *	12.66 ± 12.228 *
ACC/%	0.926 ± 0.120	0.943 ± 0.127
Updating		
RT/ms	1143.294 ± 300.018 *	1032.98 ± 198.260 *
ACC/%	0.595 ± 0.224 *	0.681 ± 0.191 *
Cognitive flexibility		
RT/ms	378.713 ± 114.807 ***	269.954 ± 70.261 ***
ACC/%	0.897 ± 0.079	0.909 ± 0.120

Note: A sample of 217 participants was included in this analysis. Descriptive data are presented as means (Ms) and standard deviations (SDs). BMI: body mass index, ACC: accuracy, RT: reaction time. * means *p* ≤ 0.05, *** means *p* ≤ 0.001.

**Table 2:** Significant changes in GMV between groups.

Regions	Mini Coordinates			Activation Cluster	Max t-Statistic
	X	Y	Z		
Right ParaHippocampal	19.5	−12	−31.5	116	4.95
Right Frontal-Mid-Orb	27	42	−16.5	315	5.76
Right Putamen	24	−4.5	9	1167	5.83
Left Putamen	−22.5	−1.5	9	938	6.30
Left Caudate	−3	6	−6	52	5.08
Left Thalamus	−3	−22.5	7.5	1003	6.42
Right Thalamus	13.5	−7.5	9	126	4.72

**Table 3:** Differences in GMV between the groups (M ± SD).

Region	Low CRF Group GMV/mm^3^	High CRF Group GMV/mm^3^	Cohen’s d
Right ParaHippocampal	0.515 ± 0.036	0.527 ± 0.049	−0.266
Right Frontal-Mid-Orb	0.428 ± 0.043 ^+^	0.456 ± 0.048 ^+^	−0.603
Right Putamen	0.524 ± 0.051	0.543 ± 0.059	−0.346
Left Putamen	0.537 ± 0.046	0.550 ± 0.057	−0.247
Left Caudate	0.405 ± 0.037	0.423 ± 0.058	−0.381
Left Thalamus	0.496 ± 0.052	0.506 ± 0.062	−0.172
Right Thalamus	0.542 ± 0.050	0.546 ± 0.060	−0.066

Note: with Bonferroni correction, ^+^ means *p* < 0.025.

**Table 4:** Indirect model test of ORBmid.R GMV.

Regression Equation (*n* = 130)		Simulation Index			Coefficient Significance	
Result Variables	Forecast Variables	*R*	*R* ^2^	*F*_(*df*)_	B	*t*
Updating RT		0.239	0.057	7.802		
	Gender				0.043	0.486
	Age				0.122	1.409
	BMI				0.043	0.479
	CRF				−0.240	−2.793 *
GMV		0.187	0.083	11.558		
	Gender				0.082	0.946
	Age				−0.115	−1.341
	BMI				−0.062	−2.459
	CRF				0.288	3.400 *
Updating RT		0.285	0.081	5.616		
	Gender				0.056	0.644
	Age				0.105	1.212
	BMI				0.018	0.202
	GMV				−0.161	−1.814 *
	CRF				−0.193	−2.177 *

Note: All variables in the model are substituted into the regression equation by the standardized variables. BMI: body mass index, GMV: gray matter volume, CRF: cardiorespiratory fitness, RT: reaction time. * means *p* ≤ 0.05.

**Table 5:** Decompositions list of total, direct and intermediate effects.

	Effect Value	Boot SE Standard Error	Boot LLCI Limit	Boot ULCI Limit	Proportion
Total effect	−0.240	0.086	−0.410	−0.070	100%
Indirect effect	−0.047	0.025	−0.101	−0.004	19.6%
Direct effect	−0.193	0.089	−0.370	−0.018	80.4%

Note: Boot Standard Error, Boot LLCI Limit, and Boot ULCI Limit respectively refer to the standard error of indirect effects estimated by the partially corrected percentile bootstrap, the lower limit of the 95% confidence.

## Data Availability

The data presented in this study are available on request from the corresponding author. The data are not publicly available due to the privacy of the participants.
